# Hypertension Control in Bangladesh: Changes, Sociodemographic Variation, and Socioeconomic Inequality from the 2017–18 to 2022 Bangladesh Demographic and Health Surveys

**DOI:** 10.5334/gh.1575

**Published:** 2026-07-27

**Authors:** Md Mostafa Monower, Shehab Uddin Al Abid, Shamim Jubayer, Mahfuzur Rahman Bhuiyan, Mohammad Abdullah Al Mamun, Sohel Reza Choudhury

**Affiliations:** 1 Department of Epidemiology & Research, National Heart Foundation Hospital & Research Institute, Dhaka, Bangladesh; 2 Nuffield Department of Population Health, University of Oxford, United Kingdom; 3 Health Data Research UK Oxford, University of Oxford, United Kingdom

**Keywords:** hypertension, blood pressure control, population-level hypertension control, hypertension care cascade, Bangladesh, South Asia

## Abstract

**Background:**

Hypertension is a major public health challenge in Bangladesh, yet population-level blood pressure control remains poorly characterized. This study estimated changes in population-level hypertension control between the Bangladesh Demographic and Health Survey (BDHS) 2017–18 and BDHS 2022 and examined sociodemographic variation and socioeconomic inequality in hypertension control.

**Methods:**

Nationally representative BDHS 2017–18 (*n* = 13,131) and BDHS 2022 (*n* = 14,296) data were analyzed using a cross-sectional design. Hypertension was defined as systolic blood pressure ≥140 mmHg, diastolic blood pressure ≥90 mmHg, or antihypertensive medication use. Population-level control was defined as blood pressure <140/90 mmHg among hypertensive individuals in the population. Adjusted control rates and sociodemographic variation were estimated using pooled survey-weighted multivariable logistic regression with marginal standardization. Socioeconomic inequality was assessed using the concentration index (CnI), slope index of inequality (SII), and relative index of inequality (RII).

**Findings:**

Among individuals with hypertension, control increased from 12.5% (95% CI: 11.3–13.8) in 2017–18 to 19.3% (17.5–21.2) in 2022, alongside increases in awareness from 42.4% (40.5–44.4) to 54.3% (51.9–56.6) and treatment from 37.0% (35.2–38.9) to 44.6% (42.1–47.0). Hypertension prevalence was 27.5% (26.5–28.5) in 2017–18 and 20.5% (19.6–21.4) in 2022. Adjusted control improved across most sociodemographic groups, with the largest increases among older adults (+6.5%), females (+6.3%), urban residents (+6.8%), individuals with higher education (+7.3%), and residents of Sylhet (+7.9%) and Chattogram (+7.2%) divisions. Socioeconomic inequality also increased between 2017–18 and 2022, with a persistent pro-rich pattern (CnI: 0.11 to 0.13; SII: 8.5% to 15.1%; RII: 1.99 to 2.22).

**Conclusion:**

Population-level hypertension control in Bangladesh improved between survey rounds, although socioeconomic and geographic disparities persisted. Differences in survey implementation and measurement methods warrant cautious interpretation of changes over time. Strengthening equitable primary care and medication access may help sustain improvements and reduce disparities.

## Graphical Abstract

**Figure:**
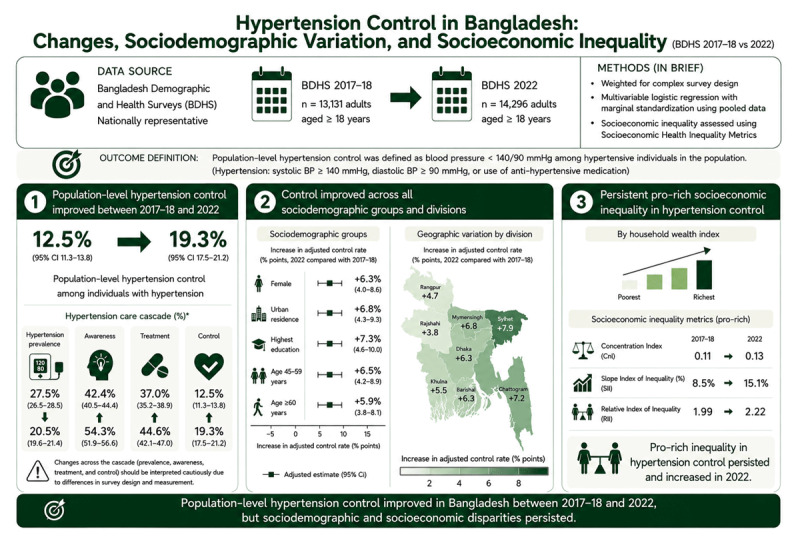

## Introduction

Hypertension is a silent epidemic and a leading contributor to the global burden of cardiovascular disease (CVD) and stroke, accounting for an estimated 10.8 million deaths globally in 2021 (1). In Bangladesh, non-communicable diseases (NCDs) now account for more than 70% of all deaths, with hypertension emerging as a major public health concern (2). Despite modest gains in awareness, treatment, and control in recent years, national control rates remain unacceptably low, particularly at the community level, where barriers in healthcare access, medication adherence, and follow-up services hinder effective management (3,4).

In response to this growing challenge, the Government of Bangladesh launched the Bangladesh Hypertension Control Initiative (BHCI) in 2018, beginning in Sylhet district and expanding to Sylhet division in 2021, with national scale-up underway by 2023 (5). Evaluations to date include programmatic reports indicating that facility-based blood pressure control in BHCI sites increased substantially, from approximately 20% in early implementation to nearly 60–64% in recent years (5,6). The BHCI incorporates evidence-based interventions, including the WHO-HEARTS technical package and updated national hypertension management guidelines, to strengthen screening, treatment, and long-term disease control (7). Core components include simplified treatment protocols, team-based care, a consistent medication supply, use of the Simple app for monitoring and follow-up, and integration of NCD services within primary healthcare (PHC) (5,8). A modeling study estimated that scaling up the WHO HEARTS program across Bangladesh could increase the national hypertension control rate from 13% to 33% and avert approximately 9,400 deaths by 2030 (9).

Assessment of national hypertension trends is important for tracking population-level progress in control (10). Facility-based control rates reflect blood pressure control among patients attending healthcare services, whereas population-level control rates assess control among hypertensive individuals identified through population-level surveys (11,12). Despite its importance, population-level control remains underexamined in low- and middle-income countries (12). The Bangladesh Demographic and Health Survey (BDHS), a nationally representative survey, provides a valuable opportunity to assess these indicators over time. This study aims to evaluate changes in population-level hypertension control between 2017–18 and 2022, examine sociodemographic variation in control, and assess socioeconomic inequality in hypertension control using inequality metrics. The findings are expected to inform ongoing national efforts, guide policy planning, and contribute to the global discourse on scalable hypertension control strategies in low- and middle-income countries.

## Methods

### Study design and data sources

This study is a secondary cross-sectional analysis of nationally representative BDHS 2017–18 and BDHS 2022 data (13,14). Both surveys were conducted by the National Institute of Population Research and Training (NIPORT), Medical Education and Family Welfare Division, Ministry of Health and Family Welfare, with technical assistance from ICF International through the DHS Program, a USAID-funded initiative supporting global health surveys. Fieldwork was implemented by Mitra and Associates. BDHS 2017–18 was conducted from October 2017 to March 2018, and BDHS 2022 from June to December 2022. Both surveys used standardized methods and collected comparable demographic, socioeconomic, and health data, allowing valid cross-survey comparisons (15,16).

### Study population and sampling design

Both surveys used a two-stage stratified cluster sampling design to ensure national representativeness across urban and rural areas, using the 2011 Population and Housing Census of the Bangladesh Bureau of Statistics as the sampling frame. In the first stage, enumeration areas (EAs) were selected using probability proportional to size (PPS). BDHS 2017–18 selected 675 EAs (250 urban, 425 rural), while BDHS 2022 selected 675 EAs (237 urban, 438 rural). In the second stage, BDHS 2017–18 selected 30 households per EA (20,250 total), and BDHS 2022 selected 45 households per EA (30,375 total) (Supplementary Figures 1 and 2). For blood pressure measurements, both surveys included adults aged ≥18 years. BDHS 2017–18 measured blood pressure in one-quarter of sampled households (5,063), yielding 13,131 eligible adults (7,427 women and 5,704 men). In BDHS 2022, 15 households per EA were selected for biomarker collection, and in half of these (approximately one-sixth of all), blood pressure was measured for all adults. The final analytical sample included 14,296 adults (7,899 women and 6,397 men) (15,16). Potential selection bias was assessed by comparing sociodemographic characteristics between included (*n* = 13,131) and excluded (*n* = 1,573) individuals in BDHS 2017–18 (Supplementary Table 6).

### Data collection

Both surveys followed standardized data collection protocols. Questionnaires were developed in English, translated into Bangla, and pretested in rural and urban areas to ensure clarity and cultural relevance. BDHS 2017–18 used paper-based interviews, while BDHS 2022 adopted Computer-Assisted Personal Interviewing (CAPI) to improve data quality. Field teams underwent rigorous training, and data collection was monitored through field supervision and random spot checks (15,16).

### Blood pressure measurement

Blood pressure was measured using validated digital monitors. BDHS 2017–18 used the LIFE SOURCE® UA-767 Plus, and BDHS 2022 used the UA-767F/FAC model. Three measurements were taken at 5-minute intervals during a single sitting. The average of the second and third readings was used; if the third was missing, the second was used alone. If both were unavailable, the first reading was used (15,16).

### Variable definition

#### Outcome variables

The primary outcome was the population-level *hypertension control*, defined as the proportion of individuals with hypertension in the population who had an average systolic blood pressure (SBP) <140 mmHg and diastolic blood pressure (DBP) <90 mmHg at the time of the survey (17,18). This indicator reflects population-level control rather than control among facility-based patients.

To reflect the care continuum, we also defined three intermediate binary outcomes (19):

1. *Hypertension prevalence*: defined as SBP ≥140 mmHg, DBP ≥90 mmHg, or current use of antihypertensive medication.
2. *Awareness*: defined as self-reported prior diagnosis of hypertension by a health professional, among hypertensive individuals.
3. *Treatment*: defined as current use of antihypertensive medication, among hypertensive individuals.

Each outcome was assessed separately for BDHS 2017–18 and BDHS 2022 to allow comparison across survey years.

#### Explanatory Variables

The explanatory variables included key sociodemographic characteristics that are known to influence hypertension management and were consistently available in both BDHS 2017–18 and BDHS 2022 datasets (Supplementary Tables 2 and 3). These variables were assessed to examine variation in hypertension control in the population and included:

- *Age group* (18–29, 30–44, 45–59, ≥60 years), categorized to distinguish younger adults, early/mid-adulthood, late working age, and older adults, and to facilitate assessment of variation in hypertension control across these age strata
- *Sex* (male, female)
- *Place of residence* (urban, rural)
- *Administrative division* (Rangpur, Rajshahi, Mymensingh, Sylhet, Dhaka, Khulna, Barishal, Chattogram arranged from north to south to reflect geographic ordering)
- *Educational attainment* (no education, primary, secondary, higher)
- *Wealth index quintile* (poorest, poorer, middle, richer, richest)

All variables were harmonized across survey rounds to ensure comparability between BDHS 2017–18 and BDHS 2022 (15,16).

### Statistical analysis

All analyses accounted for the complex survey design of the BDHS, incorporating sampling weights, clustering, and stratification to ensure nationally representative estimates. Analyses followed a complete case approach; individuals with missing outcome or covariate data were excluded. As part of the sensitivity analysis, we compared sociodemographic characteristics between individuals included and excluded from the blood pressure measurements in BDHS 2017–18 to assess potential selection bias (Supplementary Table 6). Descriptive statistics were used to summarize the background characteristics of the study population for each survey round and are presented as supplementary tables. Age, a continuous non-normally distributed variable, was summarized as weighted median and interquartile range (IQR). Hypertension care cascade indicators, including prevalence, awareness, treatment, and control, were estimated separately for BDHS 2017–18 and BDHS 2022 using survey-weighted proportions with 95% confidence intervals. To estimate adjusted hypertension control rates and changes between survey years, pooled survey-weighted multivariable logistic regression models were fitted among individuals with hypertension, with survey year included as the primary comparison variable. Models were adjusted for age, sex, education, place of residence, administrative division, and wealth index (15,16,20,21). Sociodemographic variables were coded using comparable BDHS definitions and categories across survey rounds to ensure consistency of pooled analyses. Adjusted control rates and absolute differences between survey years were estimated using marginal standardization. Age was modelled as a continuous variable, and a quadratic term was included to account for potential non-linear effects of age on hypertension control. Statistical significance was defined as *p* < 0.05. To examine socioeconomic inequality in population-level hypertension control, the concentration index (CnI), the slope index of inequality (SII), and the relative index of inequality (RII) were calculated (22). Ridit scores were generated from the weighted distribution of the household wealth index separately for each survey year. The CnI was derived using the weighted covariance between the control indicator and the ridit score, scaled by the mean prevalence of control; differences in CnI between survey years were assessed using bootstrap estimation. The SII was estimated using pooled survey-weighted linear regression models incorporating survey year and ridit score to quantify absolute inequality and its change between survey rounds. The RII was estimated using pooled survey-weighted Poisson regression models with a log link incorporating survey year and ridit score to quantify relative socioeconomic inequality and changes between survey rounds (22). Additionally, division-wise changes in adjusted population-level hypertension control between surveys were visualized using a Bangladesh administrative boundary shapefile in R to display geographic variation. Analyses were conducted using Stata version 17.0 and R version 4.3.2, with variance estimation based on Taylor linearization and robust standard errors.

### Ethical considerations

This study used de-identified secondary data from the BDHS 2017–18 and 2022, available from the DHS Program. Both surveys were implemented by the NIPORT, Ministry of Health and Family Welfare, with technical assistance from ICF. Ethical approval for data collection was obtained from the Institutional Review Board of ICF and the Bangladesh Medical Research Council (BMRC). Informed consent was obtained from all participants prior to interviews and biomarker measurements (15,16). Approval to use the BDHS datasets for this analysis was granted by the DHS Program on 11 August 2025. The authors did not have access to any information that could identify individual participants. As the analysis used anonymized secondary data, no additional ethical approval was required.

## Results

### Hypertension care cascade

**Figure 1:**
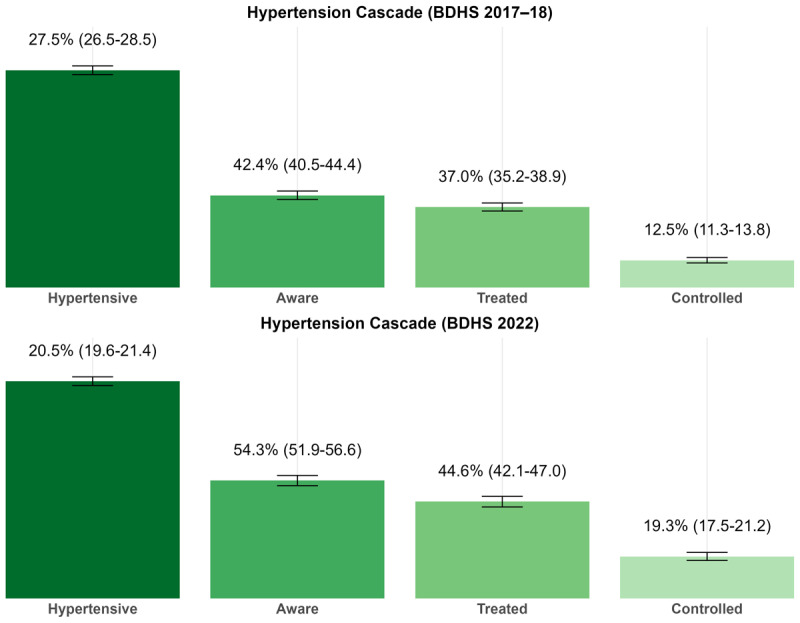
Hypertension care cascade among adults in Bangladesh, BDHS 2017–18 and 2022. Bars show weighted prevalence (% with 95% CI). Hypertension is shown for the total adult population; awareness, treatment, and control are shown among individuals with hypertension.

The median age of respondents was 36 years (IQR 26–50) in BDHS 2017–18 and 38 years (IQR 27–53) in BDHS 2022 (Supplementary Tables 2 and 3). The hypertension care cascade, based on BDHS 2017–18 and BDHS 2022, showed that the estimated prevalence of hypertension among adults was 27.5% (95% CI: 26.5–28.5) in 2017–18 and 20.5% (19.6–21.4) in 2022 (Figure 1). This lower estimate in 2022 was consistently observed across all age groups, with the largest absolute differences seen among younger adults aged 30–44 and 45–59 years (Supplementary Table 5). Mean SBP and DBP levels among individuals not receiving antihypertensive medication were also lower in 2022 compared to 2017–18 overall (SBP: 115.7 vs 119.6 mmHg; DBP: 76.0 vs 79.1 mmHg), with similar patterns observed in both urban and rural settings (Supplementary Table 4). Among hypertensive individuals (Figure 1), awareness increased from 42.4% (95% CI: 40.5–44.4) to 54.3% (95% CI: 51.9–56.6), treatment from 37.0% (95% CI: 35.2–38.9) to 44.6% (95% CI: 42.1–47.0), and control from 12.5% (95% CI: 11.3–13.8) to 19.3% (95% CI: 17.5–21.2).

Across both surveys, urban populations consistently demonstrated higher cascade performance than rural counterparts in crude control rates (Supplementary Figure 3). In 2022, hypertension control was 22.6% (95% CI: 19.3–26.2) in urban areas and 17.9% (95% CI: 15.9–20.1) in rural areas, compared with 14.9% (95% CI: 13.2–16.6) and 11.5% (95% CI: 10.1–12.9), respectively in 2017–18. In BDHS 2017–18, excluded individuals were disproportionately younger, male, urban residents, and from higher wealth quintiles (Supplementary Table 6), suggesting potential selection bias in blood pressure measurements.

### Population-level hypertension control by sociodemographic characteristics

**Figure 2:**
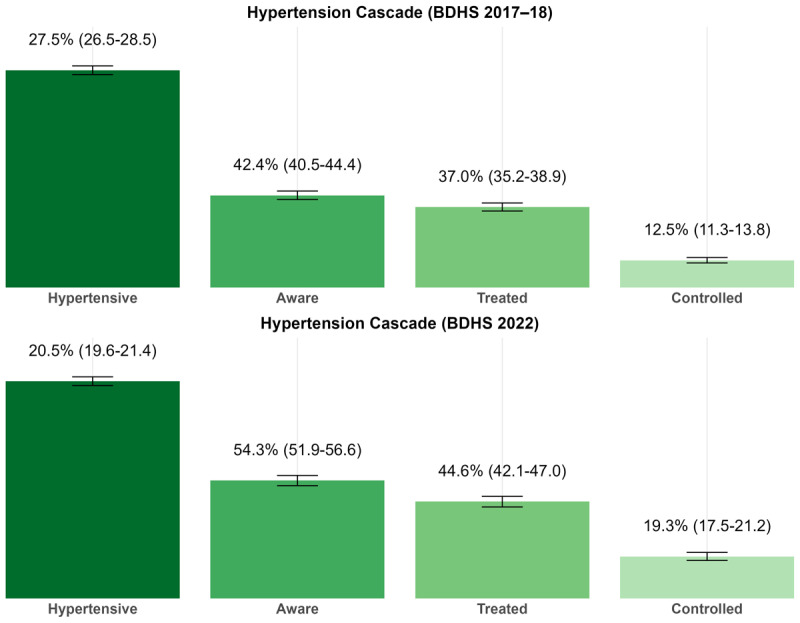
Adjusted population-level hypertension control by sociodemographic characteristics: BDHS 2017–18 and BDHS 2022. *Note*: Rates represent adjusted control percentages calculated via marginal standardization from a survey weighted pooled logistic regression model. The model adjusted for continuous age, age-squared, sex, education, residence, division, wealth quintile, and survey wave (time period). For presentation in the plot, age is displayed as a categorized variable. Total pooled sample size: N = 6,677 (n = 3,707 for BDHS 2017–18 and n = 2,970 for BDHS 2022).

Figure 2 presents adjusted population-level hypertension control rates and changes between BDHS 2017–18 and BDHS 2022 across sociodemographic groups. Overall adjusted control increased from 12.8% (95% CI: 11.6–14.1) in 2017–18 to 18.7% (95% CI: 17.0–20.5) in 2022. Control improved across all sociodemographic groups, although the magnitude of change varied. Larger increases were observed among adults aged 45–59 years (+6.5%) and ≥60 years (+5.9%), while control remained comparatively low among those aged 18–29 years in both surveys. Females had higher adjusted control rates than males in both survey rounds and experienced a larger improvement (+6.3% vs +4.9%). Urban residents showed greater gains than rural residents (+6.8% vs +5.5%).

Educational and socioeconomic differences in hypertension control were also apparent. Individuals with higher education experienced one of the largest increases in control (+7.3%), and improvements were observed across all wealth quintiles, ranging from +4.7% among the poorest to +7.3% among the richest groups.

### Geographic variation in change of population-level hypertension control

**Figure 3:**
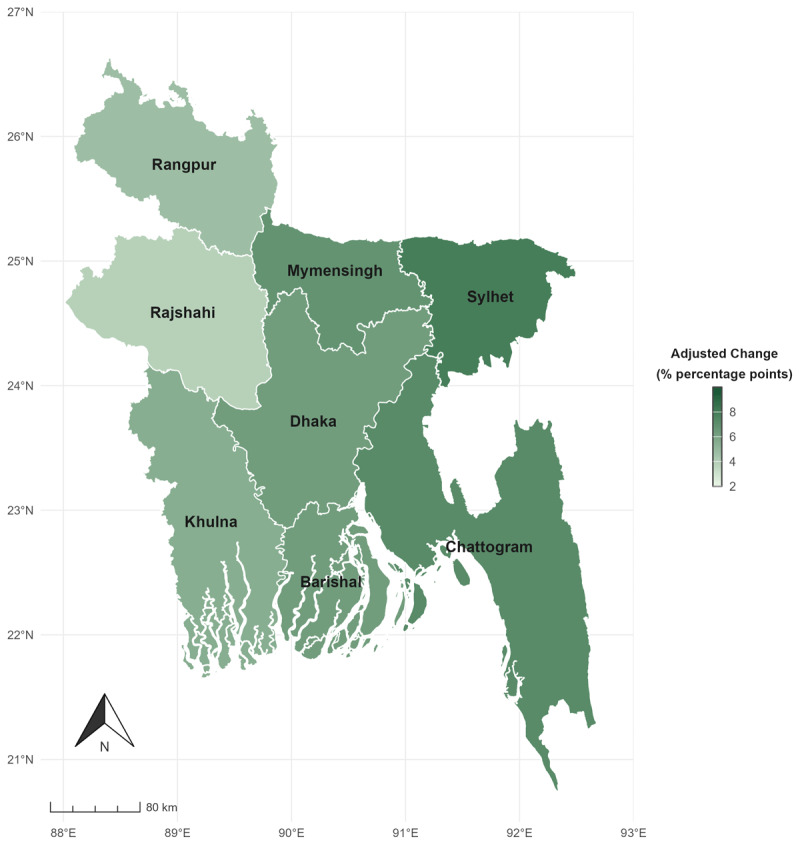
Change in population-level hypertension control in Bangladesh by division, BDHS 2017–18 to 2022. Map shows the change in control rate (%) among individuals with hypertension between the two survey rounds; darker shading indicates larger improvements.

Geographic disparities in population-level hypertension control persisted across divisions between BDHS 2017–18 and BDHS 2022. As shown in Figure 2, Sylhet division consistently recorded the highest adjusted control rate, increasing from 18.7% (95% CI: 15.1–22.3) in 2017–18 to 26.6% (95% CI: 21.9–31.2) in 2022. In contrast, Rajshahi division had the lowest adjusted control rates in both survey rounds, increasing from 7.4% (95% CI: 5.6–9.2) to 11.3% (95% CI: 8.7–13.8). Figure 3 illustrates the absolute change in control rate by division between survey rounds. The largest increase was observed in Sylhet (+7.9%; 95% CI: 4.9–10.8), followed by Chattogram (+7.2%; 95% CI: 4.5–9.9), Mymensingh (+6.8%; 95% CI: 4.2–9.4), Dhaka (+6.3%; 95% CI: 3.9–8.7), and Barishal (+6.3%; 95% CI: 3.9–8.8). Increases were also observed in Khulna (+5.5%; 95% CI: 3.4–7.7), Rangpur (+4.7%; 95% CI: 2.8–6.5), and Rajshahi (+3.8%; 95% CI: 2.3–5.4).

### Socioeconomic inequality in population-level hypertension control

Socioeconomic inequality in population-level hypertension control was evident in both survey rounds (Table 1). In BDHS 2017–18, the CnI was 0.11, the SII was 8.5% (95% CI: 4.4–12.6), and the RII was 1.99 (95% CI: 1.42–2.79), indicating a pro-rich distribution of hypertension control. Inequality widened in BDHS 2022, with a higher CnI (0.13), SII (15.1%; 95% CI: 9.0–21.3), and RII (2.22; 95% CI: 1.61–3.06). However, the differences were not statistically significant.

**Table 1:** Socioeconomic inequality in hypertension control, BDHS 2017–18 and 2022.


SURVEY YEAR	CONCENTRATION INDEX (CnI)	SLOPE INDEX OF INEQUALITY (SII)% (95%CI)	RELATIVE INDEX OF INEQUALITY (RII) (95%CI)

**BDHS 2017–18**	0.11	8.5 (4.4–12.6)	1.99 (1.42–2.79)

**BDHS 2022**	0.13	15.1 (9.0–21.3)	2.22 (1.61–3.06)

**Comparison estimate (95% CI), *p-value***	0.02 (–0.05–0.09), *p* = 0.641	6.7 (–0.7–14.0), *p* = 0.077	1.12 (0.70–1.78), *p* = 0.641


**Abbreviations:** CnI, concentration index (values >0 indicate a pro-rich distribution); SII, slope index of inequality (absolute difference in predicted control prevalence, in percentage points, between the poorest and richest); RII, relative index of inequality (prevalence ratio comparing the richest with the poorest). *p*-values derived from bootstrap-based comparison for CnI and pooled survey-weighted regression models for SII and RII. CnI and SII comparison estimates represent absolute differences; RII comparison estimates represent ratios.

## Discussion

This study examined changes in population-level hypertension control in Bangladesh between BDHS 2017–18 and BDHS 2022 using nationally representative survey data. Overall, control, awareness, and treatment were higher in 2022 than in 2017–18, although important socioeconomic and geographic disparities remained.

The estimated prevalence of hypertension was lower in BDHS 2022 compared to BDHS 2017–18. This difference should not be interpreted as a true epidemiologic decline. The age and sex distributions, including the proportion of older adults and the mean age, were similar across surveys (15,16). However, non-response to blood pressure measurement in BDHS 2017–18 was not random, with excluded individuals differing systematically by age, sex, education, residence, division, and wealth, likely leading to an overestimation of prevalence in that round, partly explaining the lower estimate observed in 2022. Additionally, the mean systolic and diastolic pressures among untreated individuals in 2022 were approximately 4 mmHg and 3 mmHg lower, respectively, reducing the number meeting the diagnostic threshold of hypertension. BDHS 2022 also used a different blood pressure device (UA-767F/FAC), replacing the UA-767 Plus used in 2017–18; device-related variation is a known source of systematic measurement bias. The shift from paper-based data collection to CAPI in 2022 may have further improved data consistency and reduced entry errors. This combination of factors likely explains the consistently lower prevalence observed across all age groups in BDHS 2022. Findings from other national sources, including WHO STEPS surveys, indicate that hypertension prevalence in Bangladesh has remained relatively stable in recent years (4,7). In the regional context, prevalence is reported at 22.6% in India, 30.1% in Bhutan, and 24.5% in Nepal (20,23,24). Taken together, these findings support the interpretation that the lower estimate observed in BDHS 2022 is most likely attributable to methodological differences, rather than a true epidemiologic decline. Therefore, prevalence estimates are not directly comparable between surveys. For assessing national progress, awareness, treatment, and control, particularly population-level control, are more informative.

In contrast to the uncertain trend in hypertension prevalence, improvements were observed in awareness, treatment, and control between BDHS 2017–18 and 2022. Among hypertensive individuals, awareness increased from 42.4% to 54.3%, treatment from 37.0% to 44.6%, and control from 12.5% to 19.3%, indicating a positive trajectory in hypertension management at the national level. Compared to the WHO STEPS 2018 survey, which reported awareness at 46.3%, treatment at 27.3%, and control at only 10.9%, the BDHS 2022 data suggest improvement in the hypertension cascade over recent years (19). While encouraging, these findings should be interpreted cautiously because of methodological differences between survey rounds. Nevertheless, these improvements remain below global benchmarks and highlight the ongoing burden of undiagnosed and poorly controlled hypertension. Bangladesh’s performance appears moderate within the South Asian context, with recent national estimates reporting control rates of 22.5% in India and 8.0% in Nepal (25,26). Within Asia, South Korea reports about 47% overall hypertension control (KNHANES 2020) and 69–73% control among treated patients (KNHANES 2008–2017), illustrating what is achievable with standardized primary care and reliable drug supply (27,28). Global evidence from GBD 2019 and PURE shows that control remains low in most low- and middle-income countries, especially among rural and socioeconomically disadvantaged groups (29,30). In contrast, Cuba and Iran have achieved much higher control through strong primary care, reliable drug supply, and structured follow-up (18,31). However, these experiences offer valuable lessons, but their application in Bangladesh will require adaptation to local health system capacity, resource availability, and population health behaviors. Persistent gaps between awareness, treatment, and control emphasize the need to strengthen continuity of care, adherence support, and structural equity.

Control was higher among older adults and women, consistent with global trends and may be attributed to greater healthcare engagement among these populations. Older individuals often have more frequent interactions with health systems due to multiple comorbidities, while women, especially in South Asia, tend to utilize health services more regularly through reproductive health programs (32,33). Educational attainment also remained a strong predictor of better hypertension control in both survey rounds, with higher control observed among individuals with higher levels of education. These gradients likely reflect disparities in health literacy, self-efficacy, and the ability to navigate healthcare systems (34). Wealth-based differences in population-level control were also evident, with higher control among wealthier groups in both surveys. Inequality metrics indicated a widening socioeconomic gradient in control between survey rounds, with a larger absolute gap (SII) and relative difference (RII). In practical terms, improvements in national averages may conceal persistent disadvantage among poorer populations. Structural barriers such as affordability of regular clinic visits, out-of-pocket medication costs, and inconsistent availability of free antihypertensive drugs in public facilities disproportionately affect lower-income populations (35). Strengthening financial protection mechanisms and ensuring consistent medication supply are essential to promote equitable hypertension management (8). These disparities reflect broader structural determinants, including variation in health literacy and primary care access, which are central to achieving equitable control of NCDs.

Geographic variation in population-level hypertension control also remained evident across divisions. Although all divisions experienced improvement between survey rounds, the magnitude of change varied considerably. The largest improvements were observed in Sylhet, Chattogram, and Mymensingh divisions. Several hypertension and NCD initiatives were active during the study period, including the BHCI by Bangladesh’s Ministry of Health and the National Heart Foundation of Bangladesh, which was initially implemented in Sylhet and subsequently expanded to additional divisions (5,6,10), population-level NCD programs led by the Asia Arsenic Network in Khulna, and the JICA-supported SHASTO program implemented in Narsingdi, Cox’s Bazar, and Dhaka City North (36,37). Differences in local health system capacity, availability of antihypertensive medication, implementation approaches, and population health-seeking behavior may contribute to this heterogeneity. However, the present analysis cannot determine whether improvements in individual divisions were attributable to specific programs or interventions. Nevertheless, the variation observed across divisions underscores the importance of sustaining progress through strengthened primary care systems and continued investment in hypertension management. Integrating hypertension services within primary care and aligning them with Universal Health Coverage may help reduce geographic disparities and improve long-term control. Such efforts would also support progress toward global targets for reducing premature mortality from NCDs (38).

### Strengths and limitations

The use of population-level hypertension control as the primary outcome strengthens the relevance of this study to population-level policy planning. Unlike facility-based or treatment-specific indicators, population-level control reflects the proportion of all hypertensive individuals whose blood pressure is controlled, regardless of care-seeking behavior. The use of two nationally representative BDHS, pooled survey-weighted analyses, and adjusted marginal prevalence estimates enabled robust comparison across survey rounds and population subgroups. This study also has several limitations. Blood pressure was measured during a single visit in each survey, which may not reflect sustained control and could misestimate true prevalence. Awareness and treatment were self-reported and subject to recall bias. The cross-sectional design limits causal inference and assessment of long-term control. Geographic analysis was restricted to administrative divisions. Differences between survey rounds, including blood pressure measurement devices, data collection methods, and non-response patterns, may have influenced both prevalence and control estimates. Therefore, the observed improvements in hypertension control should be interpreted cautiously. Finally, population-level control was assessed at a single time point and does not capture long-term adherence.

In conclusion, population-level hypertension control in Bangladesh was higher in BDHS 2022 than in BDHS 2017–18, alongside improvements in awareness and treatment. However, differences in survey implementation and measurement methods warrant cautious interpretation of changes over time. Despite overall progress, substantial socioeconomic and geographic disparities in hypertension control persisted, and socioeconomic inequality remained concentrated among wealthier population groups. Strengthening equitable access to hypertension services through PHC, improving continuity of care, and ensuring reliable medication availability will be essential to further improve hypertension control and reduce the burden of CVD in Bangladesh.

## Additional Files

The additional files for this article can be found as follows:

10.5334/gh.1575.s1 Supplementary Figure 1. Flow diagram of final sample size for BDHS 2017–18.

10.5334/gh.1575.s2 Supplementary Figure 2. Flow diagram of final sample size for BDHS 2022.

10.5334/gh.1575.s3 Supplementary Figure 3. Hypertension care cascade among adults in Bangladesh overall and by urban–rural residence, BDHS 2017–18 and 2022.

10.5334/gh.1575.s4 Supplementary Table 1. Methodological differences between BDHS 2017–18 and BDHS 2022.

10.5334/gh.1575.s5 Supplementary Table 2. Background characteristics of adult participants across the hypertension cascade, BDHS 2017–18 (*n* = 13,131).

10.5334/gh.1575.s6 Supplementary Table 3. Background characteristics of adult participants across the hypertension cascade, BDHS 2022 (*n* = 14,296).

10.5334/gh.1575.s7 Supplementary Table 4. Mean distribution of blood pressure.

10.5334/gh.1575.s8 Supplementary Table 5. Distribution of hypertension prevalence by age groups across survey.

10.5334/gh.1575.s9 Supplementary Table 6. Comparison of sociodemographic characteristics between adults included and excluded from blood pressure measurement in BDHS 2017–18.

## Data Availability

Data for this study were obtained from the publicly accessible Bangladesh Demographic and Health Survey (BDHS) 2017–18 and BDHS 2022 datasets, available through the DHS Program. The datasets can be accessed at: https://dhsprogram.com/data. Researchers must register for a free account with the DHS Program, submit a request specifying the survey datasets required, and agree to the terms of data use. As per DHS Program policy, the datasets are distributed only through their official repository and cannot be shared or deposited elsewhere. However, any qualified researcher can request access through the process described above.
