# Supplementary figures and images for: Hypertension Control in Bangladesh: Changes, Sociodemographic Variation, and Socioeconomic Inequality from the 2017–18 to 2022 Bangladesh Demographic and Health Surveys
Source: Glob Heart. 2026 Jul 27;21(1):58. doi: 10.5334/gh.1575 (PMC13426450; doi:10.5334/gh.1575)

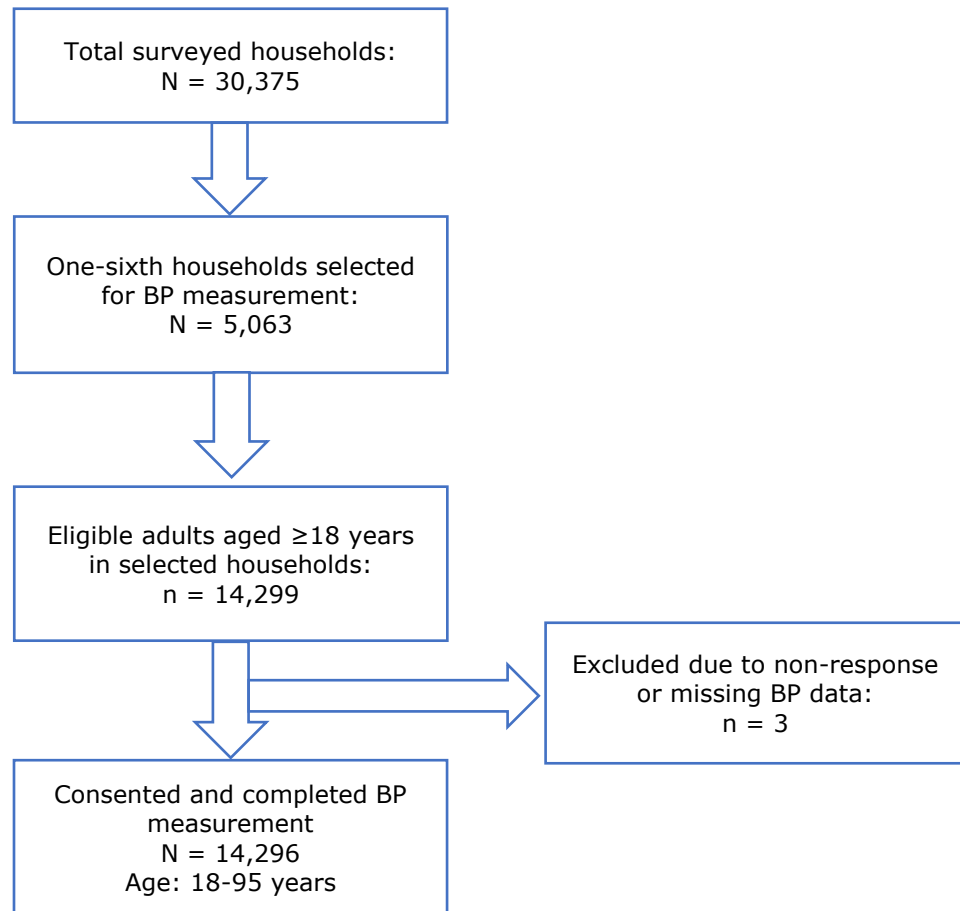

**Supplementary Figure 2.** Flow diagram of final sample size for BDHS 2022

Supplement: Supplementary Figure 2. — Flow diagram of final sample size for BDHS 2022. [file gh-21-1-1575-s2.pdf]
