# Supplementary material for: Hypertension Control in Bangladesh: Changes, Sociodemographic Variation, and Socioeconomic Inequality from the 2017–18 to 2022 Bangladesh Demographic and Health Surveys
Source: Glob Heart. 2026 Jul 27;21(1):58. doi: 10.5334/gh.1575 (PMC13426450; doi:10.5334/gh.1575)
Supplement: Supplementary Figure 3. — Hypertension care cascade among adults in Bangladesh overall and by urban–rural residence, BDHS 2017–18 and 2022. [file gh-21-1-1575-s3.pdf]

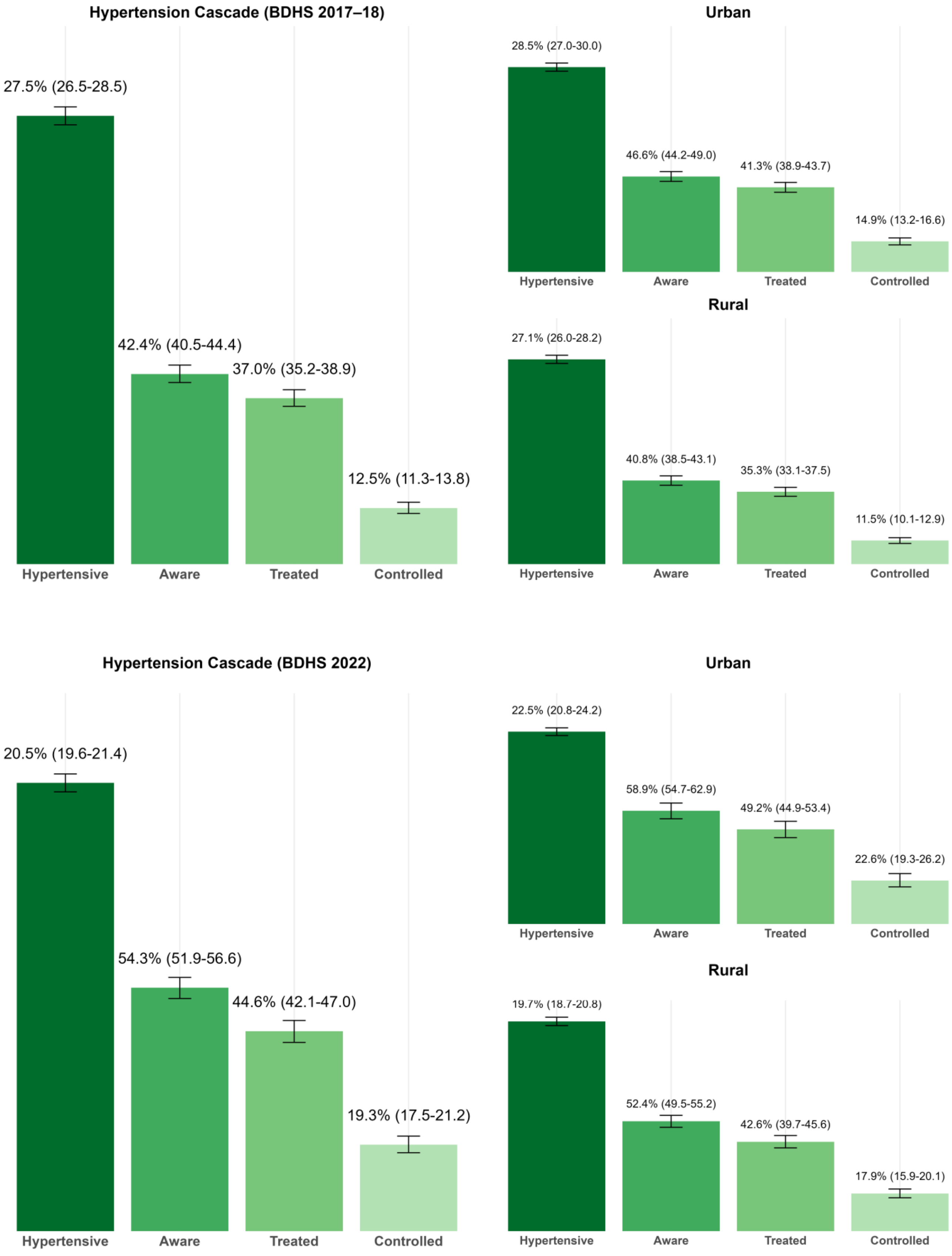

Supplementary Figure 3. Hypertension care cascade among adults in Bangladesh overall and by urban–rural residence, BDHS 2017–18 and 2022
