# Supplementary material for: Hypertension Control in Bangladesh: Changes, Sociodemographic Variation, and Socioeconomic Inequality from the 2017–18 to 2022 Bangladesh Demographic and Health Surveys
Source: Glob Heart. 2026 Jul 27;21(1):58. doi: 10.5334/gh.1575 (PMC13426450; doi:10.5334/gh.1575)
Supplement: Supplementary Table 1. — Methodological differences between BDHS 2017–18 and BDHS 2022. [file gh-21-1-1575-s4.pdf]

Supplementary Table 1. Methodological differences Between BDHS 2017–18 and BDHS 2022

Aspect	BDHS 2017–18	BDHS 2022
**Total households per enumeration area (EA)**	30 households per EA	45 households per EA
**Total selected households**	20,250 households	30,375 households
**Urban-rural EA distribution**	250 urban, 425 rural	237 urban, 438 rural
**Households selected for blood pressure measurement**	All biomarker households (7–8 per EA)	Half of the biomarker households (one-sixth of total households per EA)
**Eligibility criteria for BP measurement**	All men and women aged ≥18 years in biomarker households	All adults ≥18
**Final BP measurement sample**	13,131 adults (5,704 men, 7,427 women)	14,296 adults (6,397 men, 7,899 women)
**BP device used**	LIFE SOURCE® UA-767 Plus BP monitor	Multi-User Upper Arm Blood Pressure Monitor UA-767F/FAC
**Data collection mode**	Paper-based	Computer-Assisted Personal Interviewing (CAPI)
