# Supplementary material for: Hypertension Control in Bangladesh: Changes, Sociodemographic Variation, and Socioeconomic Inequality from the 2017–18 to 2022 Bangladesh Demographic and Health Surveys
Source: Glob Heart. 2026 Jul 27;21(1):58. doi: 10.5334/gh.1575 (PMC13426450; doi:10.5334/gh.1575)
Supplement: Supplementary Table 2. — Background characteristics of adult participants across the hypertension cascade, BDHS 2017–18 (n = 13,131). [file gh-21-1-1575-s5.pdf]

Supplementary Table 2. Background Characteristics of Adult Participants Across the Hypertension Cascade, BDHS 2017–18 (n = 13,131)

	Hypertensive		Aware among Hypertensive		Treated Among Hypertensive		Controlled Among Hypertensive		Total
	n (%)	p-value	n (%)	p-value	n (%)	p-value	n (%)	p-value	n (%)
**Age (year)**									
18-29	447 (12.3)	**<0.001**	106 (7.1)	**<0.001**	82 (6.2)	**<0.001**	52 (11.7)	0.377	4261 (32.8)
30-44	1071 (29.2)		399 (25.3)		338 (24.1)		136 (28.4)		4192 (32.0)
45-59	1079 (28.9)		542 (32.9)		486 (33.8)		156 (32.6)		2614 (19.7)
60+	1110 (29.6)		567 (34.7)		510 (35.9)		140 (27.3)		2064 (15.6)
Age (continuous)*	48 (36, 60)		52 (40, 62)		53 (42, 63)		49 (37, 60)		36 (26, 50)
**Sex**									
Male	1535 (41.2)	**0.009**	522 932.4)	**<0.001**	455 (32.0)	**<0.001**	152 (30.0)	**<0.001**	5704 (43.2)
Female	2172 (58.8)		1092 (67.6)		961 (68.0)		332 (70.0)		7427 (56.8)
**Education**									
No education	1188 (33.7)	**<0.001**	531 (34.4)	0.179	472 (35.5)	0.054	129 (28.1)	0.099	3256 (25.8)
Primary	1099 (29.2)		499 (30.3)		434 (29.8)		155 (31.4)		3946 (29.8)
Secondary	894 (24.6)		376 (24.0)		331 (24.1)		123 (27.2)		3759 (29.3)
Higher	526 (12.6)		208 (11.3)		179 (10.6)		77 (13.2)		2170 (15.1)
**Residence**									
Rural	2297 (71.6)	0.190	945 (68.8)	**0.006**	820 (68.2)	**0.002**	273 (66.1)	**0.015**	8355 (72.6)
Urban	1410 (24.4)		669 (31.2)		596 (31.8)		211 (33.9)		4776 (27.4)
**Division**									
Rangpur	508 (13.3)	**<0.001**	180 (10.4)	**0.005**	143 (9.6)	**<0.001**	39 (7.9)	**<0.001**	1625 (12.0)
Rajshahi	470 (14.2)		183 (13.0)		144 (11.7)		32 (7.6)		1689 (14.1)
Mymensingh	363 (7.1)		156 (7.0)		145 (7.4)		65 (10.8)		1501 (8.3)
Sylhet	409 (6.2)		206 (7.1)		194 (7.7)		77 (8.8)		1558 (6.5)
Dhaka	429 (20.5)		198 (22.0)		176 (22.7)		61 (22.1)		1797 (24.0)
Khulna	543 (13.3)		246 (13.8)		210 (13.2)		58 (9.9)		1791 (12.3)
Barishal	447 (6.6)		206 (7.0)		183 (7.0)		61 (7.2)		1376 (5.6)
Chattogram	538 (18.8)		239 (13.7)		221 (20.8)		91 (25.7)		1794 (17.3)
**Wealth Index**									
Poorest	636 (16.2)	**<0.001**	196 (12.6)	**<0.001**	169 (12.3)	**<0.001**	57 (11.7)	**0.003**	2517 (19.0)
Poorer	684 (18.8)		240 (15.4)		201 (14.7)		67 (14.3)		2479 (19.6)
Middle	691 (19.1)		300 (19.6)		264 (20.0)		85 (18.9)		2555 (20.4)
Richer	725 (20.1)		347 (22.1)		303 (22.0)		105 (24.7)		2563 (19.8)
Richest	872 (25.7)		531 (30.3)		479 (31.0)		170 (30.4)		3017 (21.2)
**National**	3707 (27.5)		1614 (42.4)		1416 (37.0)		484 (12.5)		13131 (100)

*Continuous non-normally distributed variables are presented as weighted median (IQR).
Reported p-values are based on Chi-squared tests.
Proportions are weighted proportion.
Abbreviations: IQR, Interquartile range; n, number of participants; %, percentage.
