# Supplementary material for: Hypertension Control in Bangladesh: Changes, Sociodemographic Variation, and Socioeconomic Inequality from the 2017–18 to 2022 Bangladesh Demographic and Health Surveys
Source: Glob Heart. 2026 Jul 27;21(1):58. doi: 10.5334/gh.1575 (PMC13426450; doi:10.5334/gh.1575)
Supplement: Supplementary Table 3. — Background characteristics of adult participants across the hypertension cascade, BDHS 2022 (n = 14,296). [file gh-21-1-1575-s6.pdf]

Supplementary Table 3. Background Characteristics of Adult Participants Across the Hypertension Cascade, BDHS 2022 (n = 14,296)

	Hypertensive		Aware among Hypertensive		Treated Among Hypertensive		Controlled Among Hypertensive		Total
	n (%)	p-value	n (%)	p-value	n (%)	p-value	n (%)	p-value	n (%)
**Age (year)**									
18-29	222 (7.0)	**<0.001**	68 (3.7)	**<0.001**	47 (3.1)	**<0.001**	26 (4.1)	**<0.001**	4280 (29.7)
30-44	779 (26.5)		360 (21.8)		283 (20.7)		138 (22.1)		4574 (32.2)
45-59	966 (32.3)		612 (36.4)		510 (36.4)		227 (37.2)		3074 (21.5)
60+	1003 (34.2)		632 (38.1)		549 (39.8)		219 (36.6)		2368 (16.6)
Age (continuous)*	52 (40, 63)		55 (44, 65)		55 (45, 65)		54 (43, 63)		38 (27, 53)
**Sex**									
Male	1092 (37.0)	**<0.001**	545 (33.5)	**<0.001**	449 (33.0)	**<0.001**	219 (37.7)	**0.726**	6397 (44.7**)**
Female	1878 (63.0)		1127 (66.5)		940 (67.0)		391 (62.3)		7899 (55.3)
**Education**									
No education	1059 (37.7)	**<0.001**	594 (37.4)	0.863	488 (37.5)	0.143	183 (31.0)	**<0.001**	3649 (26.2)
Primary	727 (24.2)		410 (24.3)		333 (23.0)		139 (22.1)		3600 (25.5)
Secondary	777 (25.2)		439 (24.9)		364 (24.9)		172 (27.2)		4271 (33.3)
Higher	407 (12.8)		229 (13.3)		204 (14.6)		116 (19.6)		2326 (16.3)
**Residence**									
Rural	1812 (70.4)	**0.006**	967 (67.9)	**0.012**	788 (67.3)	**0.013**	335 (65.3)	**0.019**	1812 (73.0)
Urban	1158 (29.6)		705 (32.1)		601 (32.7)		275 (34.7)		1158 (27.0)
**Division**									
Rangpur	397 (12.3)	**0.006**	198 (10.9)	0.089	155 (10.2)	**0.001**	72 (10.9)	**0.003**	1774 (12.0)
Rajshahi	440 (15.8)		244 (15.0)		191 (13.5)		63 (10.0		1833 (13.6)
Mymensingh	282 (6.7)		149 (6.2)		114 (5.7)		50 (6.0)		1634 (8.3)
Sylhet	342 (6.3)		226 (7.5)		198 (8.0)		95 (9.1)		1749 (6.8)
Dhaka	373 (22.0)		208 (22.3)		172 (22.1)		77 (23.1)		1927 (23.0)
Khulna	423 (13.3)		244 (13.7)		211 (14.2)		93 (13.9)		1850 (12.2)
Barishal	295 (5.6)		165 (5.9)		140 (6.1)		60 (5.8)		1524 (6.2)
Chattogram	418 (18.0)		238 (18.6)		208 (20.1)		100 (21.1)		2005 (17.9)
**Wealth Index**									
Poorest	443 (13.4)	**<0.001**	214 (11.2)	**<0.001**	161 (10.1)	**<0.001**	69 (10.4)	**<0.001**	443 (17.7)
Poorer	510 (17.6)		256 (15.6)		204 (14.8)		79 (13.4)		510 (20.1)
Middle	591 (20.4)		320 (19.6)		261 (19.2)		102 (17.4)		591 (20.2)
Richer	658 (22.2)		388 (23.1)		327 (23.6)		153 (23.3)		658 (21.0)
Richest	768 (26.3)		494 (30.6)		436 (32.3)		207 (35.5)		768 (21.1)
**National**	2970 (20.5)		1672 (54.3)		1389 (44.6)		610 (19.3)		14296 (100)

*Continuous non-normally distributed variables are presented as weighted median (IQR).
Reported p-values are based on Chi-squared tests.
Proportions are weighted proportion.
Abbreviations: IQR, Interquartile range; n, number of participants; %, percentage.
