# Supplementary material for: Hypertension Control in Bangladesh: Changes, Sociodemographic Variation, and Socioeconomic Inequality from the 2017–18 to 2022 Bangladesh Demographic and Health Surveys
Source: Glob Heart. 2026 Jul 27;21(1):58. doi: 10.5334/gh.1575 (PMC13426450; doi:10.5334/gh.1575)
Supplement: Supplementary Table 4. — Mean distribution of blood pressure. [file gh-21-1-1575-s7.pdf]

Supplementary Table 4. Mean distribution of blood pressure

Blood pressure	BDHS 2017–18			BDHS 2022		
	Urban	Rural	Total	Urban	Rural	Total
**Mean Systolic BP**	119.1 (118.2-119.8)	119.8 (119.2-120.3)	119.6 (119.1-120.0)	115.4 (114.6-116.1)	115.8 (115.3-116-3)	115.7 (115.2-116.1)
**Mean Diastolic BP**	79.6 (79.1-80.0)	79.0 (78.7-79.3)	79.1 (78.9-79.4)	76.6 (76.1-77.0)	75.8 (75.5-76.1)	76.0 (75.7-76.3)

* Weighted mean blood pressure among individuals not receiving anti-hypertensive medication
