# Supplementary material for: Hypertension Control in Bangladesh: Changes, Sociodemographic Variation, and Socioeconomic Inequality from the 2017–18 to 2022 Bangladesh Demographic and Health Surveys
Source: Glob Heart. 2026 Jul 27;21(1):58. doi: 10.5334/gh.1575 (PMC13426450; doi:10.5334/gh.1575)
Supplement: Supplementary Table 5. — Distribution of hypertension prevalence by age groups across survey. [file gh-21-1-1575-s8.pdf]

Supplementary Table 5. Distribution of hypertension prevalence by age groups across survey

Age groups	BDHS 2017–18<br>% (95%CI)	BDHS 2022<br>% (95%CI)
**18-29**	3.4 (3.0-3.8)	1.4 (1.2-1.7)
**30-44**	8.0 (7.5-8.6)	5.4 (5.0-5.9)
**45-59**	8.0 (7.4-8.5)	6.7 (6.1-7.1)
**60+**	8.1 (7.6-8.7)	7.0 (6.5-7.5)
**Total**	27.5 (26.5-28.5)	20.5 (19.6-21.4)

Weighted prevalence with 95% confidence interval
