# Supplementary material for: Hypertension Control in Bangladesh: Changes, Sociodemographic Variation, and Socioeconomic Inequality from the 2017–18 to 2022 Bangladesh Demographic and Health Surveys
Source: Glob Heart. 2026 Jul 27;21(1):58. doi: 10.5334/gh.1575 (PMC13426450; doi:10.5334/gh.1575)
Supplement: Supplementary Table 6. — Comparison of sociodemographic characteristics between adults included and excluded from blood pressure measurement in BDHS 2017–18. [file gh-21-1-1575-s9.pdf]

Supplementary Table 6. Comparison of sociodemographic characteristics between adults included and excluded from blood pressure measurement in BDHS 2017–18

	Included N = 13,131 n (%)	Excluded N=1,573 n (%)	P value
**Age**			
18-29	4261 (32.8)	606 (39.0)	**<0.001**
30-44	4192 (32.0)	427 (26.3)	
45-59	2614 (19.7)	278 (17.9)	
60+	2064 (15.6)	262 (16.7)	
**Sex**			
Male	5704 (43.2)	986 (63.4)	**<0.001**
Female	7427 (56.8)	587 (37.6)	
**Education**			
No education	3256 (25.8)	353 (23.9)	**<0.001**
Primary	3946 (29.8)	389 (25.0)	
Secondary	3759 (29.3)	429 (28.4)	
Higher	2170 (15.1)	409 (22.7)	
**Residence**			
Rural	8355 (72.6)	839 (59.5)	**<0.001**
Urban	4776 (27.4)	734 (40.5)	
**Division**			
Rangpur	1625 (12.0)	114 (6.7)	**<0.001**
Rajshahi	1689 (14.1)	148 (10.3)	
Mymensingh	1501 (8.3)	143 (6.4)	
Sylhet	1558 (6.5)	209 (6.5)	
Dhaka	1797 (24.0)	358 (34.9)	
Khulna	1791 (12.3)	158 (8.4)	
Barishal	1376 (5.6)	165 (5.4)	
Chattogram	1794 (17.3)	278 (21.4)	
**Wealth Index**			
Poorest	2517 (19.0)	260 (16.2)	**<0.001**
Poorer	2479 (19.6)	185 (12.0)	
Middle	2555 (20.4)	245 (17.1)	
Richer	2563 (19.8)	266 (17.8)	
Richest	3017 (21.2)	617 (36.9)	
**National**	13131 (89.1)	1573 (10.9)	

Reported p-values are based on bivariate Chi-squared tests.
Proportions are weighted proportion
